# PAU/GNSS-R: Implementation, Performance and First Results of a Real-Time Delay-Doppler Map Reflectometer Using Global Navigation Satellite System Signals

**DOI:** 10.3390/s8053005

**Published:** 2008-05-06

**Authors:** Juan Fernando Marchan-Hernandez, Adriano Camps, Nereida Rodriguez-Alvarez, Xavier Bosch-Lluis, Isaac Ramos-Perez, Enric Valencia

**Affiliations:** Remote Sensing Lab, Dept. Teoria del Senyal i Comunicacions, Campus Nord D3, Universitat Politecnica de Catalunya, 08034 Barcelona, Spain

**Keywords:** GPS reflectometry, Delay-Doppler Maps (DDM), sea state, digital design, embedded system, real-time, field-programmable gate array (FPGA)

## Abstract

Signals from Global Navigation Satellite Systems (GNSS) were originally conceived for position and speed determination, but they can be used as signals of opportunity as well. The reflection process over a given surface modifies the properties of the scattered signal, and therefore, by processing the reflected signal, relevant geophysical data regarding the surface under study (land, sea, ice…) can be retrieved. In essence, a GNSS-R receiver is a multi-channel GNSS receiver that computes the received power from a given satellite at a number of different delay and Doppler bins of the incoming signal. The first approaches to build such a receiver consisted of sampling and storing the scattered signal for later post-processing. However, a real-time approach to the problem is desirable to obtain immediately useful geophysical variables and reduce the amount of data. The use of FPGA technology makes this possible, while at the same time the system can be easily reconfigured. The signal tracking and processing constraints made necessary to fully design several new blocks. The uniqueness of the implemented system described in this work is the capability to compute in real-time Delay-Doppler maps (DDMs) either for four simultaneous satellites or just one, but with a larger number of bins. The first tests have been conducted from a cliff over the sea and demonstrate the successful performance of the instrument to compute DDMs in real-time from the measured reflected GNSS/R signals. The processing of these measurements shall yield quantitative relationships between the sea state (mainly driven by the surface wind and the swell) and the overall DDM shape. The ultimate goal is to use the DDM shape to correct the sea state influence on the L-band brightness temperature to improve the retrieval of the sea surface salinity (SSS).

## 1. Introduction

Remote sensing sensors allow retrieving geophysical parameters that are interesting from both scientific and commercial points of view, such as sea surface salinity, soil moisture, sea state or altimetry products. On the other hand, Global Navigation Satellite Systems (GNSS) cover the Earth with their navigation signals, used nowadays in a wide range of everyday situations, such as fleet management, vehicle guidance or leisure/outdoors interactive maps. However, as first proposed in [1] nearly fifteen years ago, these navigation signals can also be received and processed after reflecting on a certain surface to retrieve altimetry information. This approach, in short known as GNSS-R, allows inexpensively and remotely sensing other geophysical parameters over wide areas of the Earth's surface [2-4]. This is possible because the scattering mechanism ‘watermarks’ the signal so that the geophysical information regarding the reflecting surface is added to it. A very straightforward example is that of the soil moisture retrieval. The water content is related to the soil dielectric constant, which is related to the reflection coefficient. Therefore, the ratio between the incident and the reflected power of the GNSS signal can be indirectly linked to the soil moisture content.

To the present day only the GPS constellation has been fully deployed, and therefore mostly all the GNSS-R receivers built so far rely on that particular system (other GNSS are the Russian GLONASS or the European GALILEO). More specifically, the Standard Positioning Service (SPS) GPS signal broadcast at the L1 frequency (1575.42 MHz) has the following expression [5]:

(1) $$S_{L1,\text{CA}}(t)=\text{CA}(t)D(t)\sin(2\pi f_{c}t),$$

where *CA(t)* is a pseudo-random code unique to each satellite, *D(t)* is a low-speed navigation data signal, and *f_c_* is the carrier frequency. The relative motion between emitter and receiver shifts the carrier frequency of the received signal:

(2) $$S_{\text{rec}}=\text{CA}(t)D(t)\sin(2\pi (f_{c}+f_{d})t),$$

being *f_d_* the value of this Doppler frequency shift. The advantage of the instrument described in this work is that the observable is the whole Delay-Doppler Map (DDM), which is the correlation of the incoming signal with a replica of the CA code for several Doppler and code values^1^:

(3) $$\text{DDM}(f_{d},\tau )=\int_{0}^{T_{i}}(S_{a,\text{rec}}(t))\cdot \text{CA}(t+\tau )\cdot e^{-j2\pi f_{d}t}\cdot dt=\int_{0}^{T_{i}}(I(t)+j\cdot Q(t))\cdot \text{CA}(t+\tau )\cdot (\cos\left(2\pi \left|f_{d}\right|t\right)\mp j\cdot \text{sen}\left(2\pi \left|f_{d}\right|t)\right)\cdot dt,$$

where the analytic signal *S_a,rec_* = *I* + *jQ* has been used. In radar terminology the DDM is known as the Woodward ambiguity function [6]. The DDM is a complex two-dimensional function, and this presents some implications when trying to average several DDMs.

The navigation data signal *D*(*t*) (Eqns.1 and 2) has a data rate of 50 bit/s; i.e, every 20 ms there may be a polarity inversion on the received signal due to the transmission of a navigation bit. This may cause the GPS signal not to be detected if not taken into account properly. Usually the GPS receivers decode the navigation message, and are able to determine the position of this sign transition. However, in this GNSS-R implementation the navigation message is not decoded. That means that 1 out of 20 1 ms-based DDMs may be erroneous. This should not be an issue, but whenever coherently averaging a number of DDMs to improve the SNR, a common situation whenever working with low SNR GNSS-R signals, the sign change may cause an overall SNR decrease after integration. A rule-of-thumb coherence time for the sea surface at L-band is on the order of 5-10 ms [7], although it can be larger for calm seas. Therefore, an upper bound for the number of DDM to be coherently added will be between 5 and 10. In addition to this, the incoherent (module) average can last for a longer time, provided that the code and Doppler shifts due to the relative motion between emitter and receiver are accounted for.

The emitted GPS signal is right-hand circularly polarized (RHCP). However, after reflecting on the sea surface, it becomes mostly left hand circularly polarized (LHCP), with a certain degree of ellipticity due to the difference between the vertical and horizontal reflection coefficients. The PAU receiver antennas [8] are sensitive to both the horizontal and vertical linear polarizations, and it is the digital receiver that composes the LHCP from the vertical and horizontal ones. Therefore, it is of capital importance to undertake a phase and amplitude calibration before composing the required polarization.

The PAU system [8, 9] operates completely with digitized signals. The sampling frequency determines how many samples constitute the minimum ‘data strip’ to be used. Because the length of the C/A codes is 1 ms, and the sampling frequency for the whole of the PAU system [8] is 5.745 MHz, each data strip has 5745 samples, sampled at 1 bit^2^. This severe quantification of the input signal has a little effect on the GPS signal, just a small loss in signal-to-noise ratio (SNR)^3^, while it allows for a much simpler design.

From Eqn. 3 it is clear that each delay-Doppler coordinate of a DDM can be obtained from the combination and accumulation of four *partial products*:

(4a) $$\text{DDM}=\int_{T_{\mathrm{int}}}\left(\text{CA}\cdot I\cdot \mathrm{COS}+\text{CA}\cdot Q\cdot \mathrm{SIN}+j\left(\text{CA}\cdot Q\cdot \mathrm{COS}-\text{CA}\cdot I\cdot \mathrm{SIN}\right)\right)\cdot dt,fd>0\text{Hz}$$

(4b) $$\text{DDM}=\int_{T_{\mathrm{int}}}\left(\text{CA}\cdot I\cdot \mathrm{COS}-\text{CA}\cdot Q\cdot \mathrm{SIN}+j\left(\text{CA}\cdot Q\cdot \mathrm{COS}+\text{CA}\cdot I\cdot \mathrm{SIN}\right)\right)\cdot dt,fd<0\text{Hz}$$

where *CA* stands for the GPS C/A code, *I*/*Q* for the in-phase & quadrature signal components, and *SIN*/*COS* for the demodulating tones at the Doppler frequency.

The Very High Definition Level digital logic description language (VHDL) approach generates the *C*/*A* codes corresponding to all the delay coordinates, the demodulating tones associated to all the Doppler coordinates, and then combines them with the I/Q inputs to form the four *partial products* (Eqn. 4) needed to compute the complex DDM.

The goal of implementing this reflectometer-DDM generator [8, 10] is to obtain quantitative relationships between the sea state (mainly driven by the surface wind and the swell) and the overall DDM shape. It is expected that this would make possible to correct the sea state influence on the L-band brightness temperature to improve the retrieval of the sea surface salinity (SSS).

This work is divided as follows: Section 2 reviews thoroughly the system architecture, whereas section 3 deals with the preliminary results. Finally, section 4 addressees the conclusions and future research lines of the present work.

## 2. GNSS-R Reflectometer Architecture

Most of the existing GNSS-R receiver architectures just measure the correlation peak or the cut in the delay axis of the DDMs once the Doppler frequency has been compensated, which is called a ‘waveform’. This section describes the architecture of an FPGA-based real-time GPS reflectometer that computes the full two-dimensional DDM every 1 ms and performs the coherent and incoherent averaging. The core of the system is a full-custom designed DDM generator that interacts with other in-house and off-the-shelf VHDL cores.

**Figure 1.**
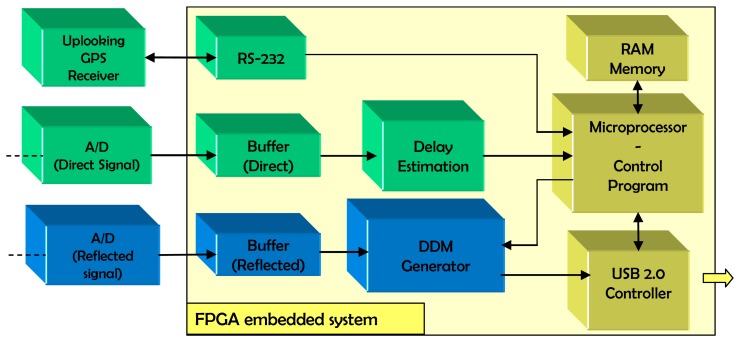
Data paths in the implemented reflectometer.

The main dataflow path of the system is that of the reflected GPS signal (Fig. 1). The PAU-RAD [11] section of the instruments sends the digitized and quantized I/Q raw data corresponding to the particular beams composed (1 or 4) to a buffer located in the reflectometer FPGA. This buffer plays a key role in the system, since it allocates the two I/Q 5745-sample strips for the up to 4 selected beams, to allow them to be processed at a higher clock rate using hardware reuse techniques. The buffer is based in two RAM-like registers that change their respective inputs and output connections. One is being written while the other is being read to generate the DDM. When the input register is full, it is swapped with the other one. From here the data is sent in blocks of 1 ms (5745 samples at the sampling frequency of the system corresponding to the period of the C/A code) to the DDM generator. Then, once the new DDM is ready, it is sent out of the system to a terminal computer, where it will be processed and stored. The transfer of up to four complex-valued DDMs every 1 ms is performed using the USB 2.0 protocol. The resulting throughput is 2 (real and imaginary parts) times 32 bits per point times 4 beams times 16 x 16 points = 8 Kbytes every 1 ms, that is 7.8 MB/s. In fact, in addition to the DDM data, the time tag of the raw data and the delay and Doppler center coordinates used for its computation are transmitted as well, since not all of the 32 bytes are used to code the DDM point values. Whenever the first of the 4 DDMs has been successfully received by the host computer, an interrupt is issued in the reflectometer system by the USB FPGA controller and the parameters for the generation of the next DDM are transferred to the DDM generator, which starts the computation using the data of the corresponding beam. After 5745 clock cycles (1 ms of data) the DDM generator activates the ‘data_ready’ signal. The microprocessor program asks the USB controller to read the DDM values from the output interface of the DDM generator, and to send these values to the host PC, closing the cycle of processing and data transfer. The integration time can be freely configured, since every 1 ms a new DDM is obtained. It is up to the receiving program to accumulate as many of them as configured, either coherently (in amplitude and phase) or incoherently (in absolute values).

The direct signal from the satellite is also processed to obtain an accurate estimate of the signal delay. To do so an upwards-looking GPS antenna receives the signal and feeds it into an A/D converter. A second buffer stores these data until they are sent to a ‘delay offset’ block that estimates the delay offset.

A third dataflow path is that of the data packets generated by a commercial GPS receiver. They enter the FPGA using an RS-232 interface using the GPS-standard TSIP protocol, and provide the system with parameters of interest such as the elevation, azimuth, power level, and Doppler of the visible satellites, simplifying to a large extent the FPGA design.

### 2.1. Hardware Setup

**Figure 2.**
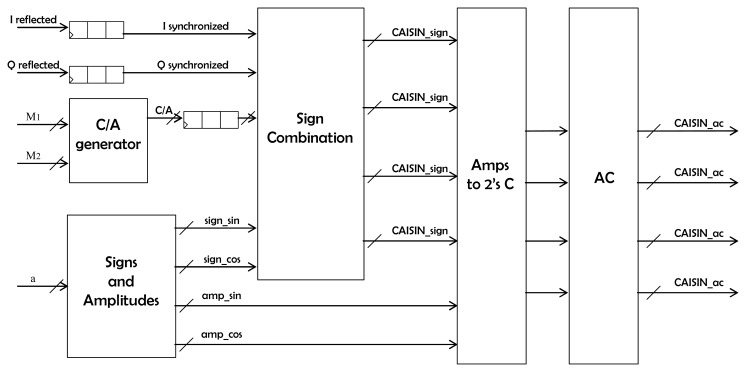
Delay Doppler Map (DDM) generator architecture. The ‘Amps to 2C’ block performs the two's complement encoding, whereas the ‘AC’ block accumulates the inputs.

**Figure 3.**
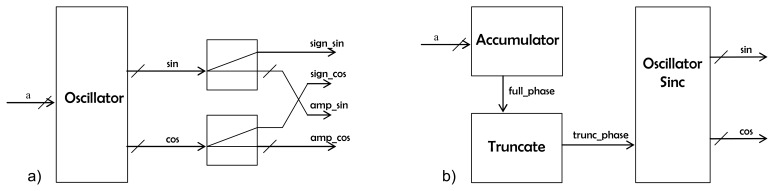
Detail of the main cores that compose the DDM generator: (a) ‘Sign and amplitudes’ block in Fig. 2 and (b) ‘Oscillator block’ in Fig. 3a.

**Figure 4.**
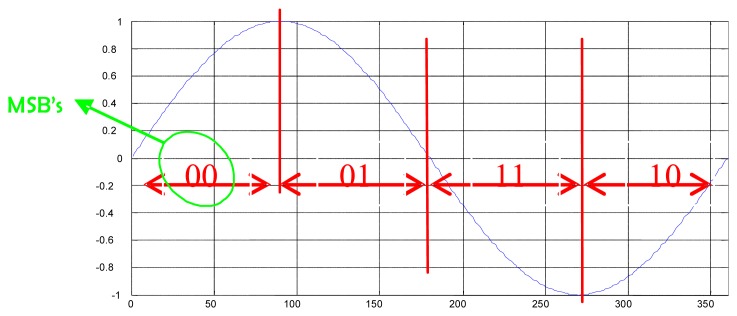
Phase MSBs role on the wave generation.

The reflectometer system has been implemented using the AVNet VIRTEX-4 LX 60 development board that includes the FPGA core, SDRAM RAM (32 MB), Flash ROM (8 MB), an UART serial interface, an USB 2.0 serial interface, an Ethernet connection, an OLED display and several other connectors. Inside the FPGA a microprocessor, data bus and RAM system is synthetized. Several peripherals are attached to the bus [10]. The most relevant is the DDM generator, which performs the Doppler compensation and the integration (Eqn. 3). The core of this generator is shown in Fig. 2. It is a synchronous block that receives the I/Q samples in a serial fashion, as well as the Doppler values and C/A code samples for all the DDM points. The outcome are the four *partial products* (Eqn. 4) that are conveniently added or subtracted (depending on the sign of the Doppler shift) and accumulated during 5745 clock cycles (1 ms of data) to obtain the real and imaginary parts of each DDM coordinate. In Fig. 2 it is seen that the I, Q and C/A code inputs are delayed by means of shift registers so that they are synchronized with the demodulating tones generated in the block *signs_and_amplitudes* (Fig. 3a). The values fed to this component determine the Doppler frequencies that are generated (they are the multiplying factors of the base frequency of the block), and thus the sign (1 bit) and amplitude (7 bits) of each frequency are obtained. Then, the block *signs_combinations* generates, for each DDM point, the sign of the four *partial products* that compose it (*CA.I.SIN, CA.I.COS, CA.Q.SIN* and *CA.Q.COS*). The core of this component is the *oscillator* block (Fig. 3b), that every clock cycle computes one sine and one cosine sample, at a frequency set by the input value *α*. To do so every clock cycle the value of the variable *full_phase* is increased in *α* units (Fig. 3b). The 9 most significant bits (*trunc_phase*) out of 18 are then fed into the *oscillator_sinc* block to generate the *sin* and *cos* outputs. This allows having a smaller frequency step without having to use a large number of bits to quantify the phase that is converted into sine/cosine amplitude. The *oscillator_sinc* component is located at the bottom of the hierarchy level. To efficiently generate the sine and cosine values, it has been taken into account that the output signal shall be quantized with a finite number of levels. The first Most Significant Bit (MSB) tells whether the sine value is positive or negative, whereas the second MSB has information on the slope sign (Fig. 4). The remaining 7 bits indicate the position within the first wave quarter. Instead of dividing the amplitude in equal-length intervals, the phase domain has been divided in such a way, so that the 7-bit phase of the wave quarter have a linear correspondence with the 7 bits that codify the amplitude. Therefore, if no further correction were made a triangular wave would be obtained. A correction for each level (from 0 to 127) that minimizes the error when comparing it with an ideal quantized sine has been computed, and it is then added to it. At the same time, the cosine is obtained by negating the 7 LSB's of the truncated phase, thus obtaining the complementary level:

(5) $${\text{Level}}_{\cos}=127-{\text{Level}}_{\sin\;},$$

The cosine sign (the MSB of *cos*) is computed as the exclusive OR of the sine's two MSB:

(6) $${\text{MSB}}_{\cos}={\text{MSB}}_{\sin\;}\text{xor}{\left(\text{MSB}-1\right)}_{\sin\;},$$

The sine and cosine waveforms are generated with 8-bit, since the preliminary simulations indicated that using fewer bits resulted in a severe DDM deformation.

**Figure 5.**
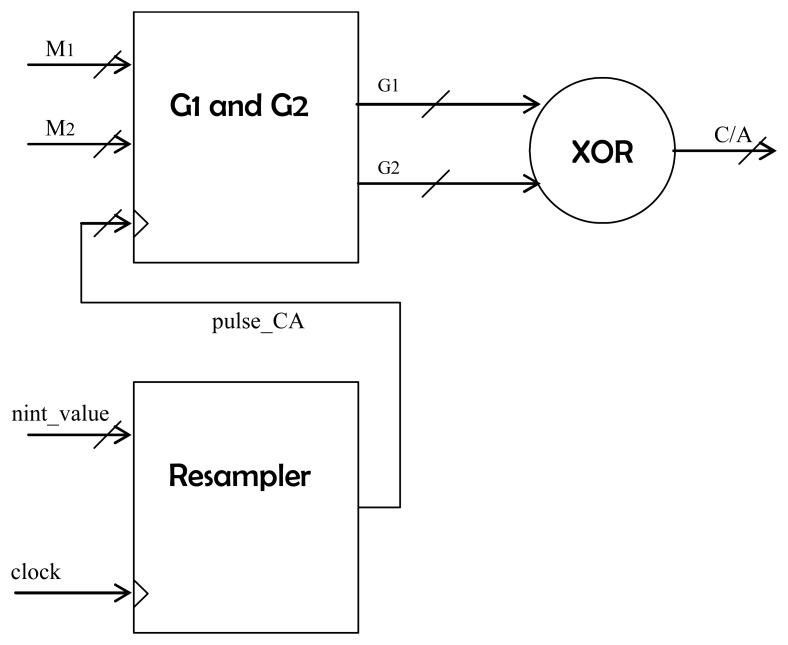
Sketch of the *C/A generator* block. The *Resampler* component drives the Linear Feedback Shift Registers (LFSR) inside the block *G1 and G2,* out of where the C/A code is obtained. The values of M1, M2 and nint_value determine the code offset and the satellite ID.

The simultaneous generation of several different-delay C/A codes is achieved with the block *CA_generator* (Figs. 2 and 5). As explained in [5], these codes are the product of two Maximum Length Sequences (MLS) *G_1_* and *G_2_* obtained using their respective linear feedback shift registers (LFSR). The satellite unique C/A code is determined by the delay of *G_2_* with respect to the non-delayed *G_1_*. Therefore, to obtain a C/A code delayed n chips, it is necessary to generate a *G_1_* sequence delayed n chips and also a *G_2_* sequence delayed n chips plus the offset that determines the satellite ID. To obtain these delayed sequences the 10-bit *M_1_* and *M_2_* masks determine the values of the *G_1_* and *G_2_* registers that are used to obtain the delayed outputs. These masks are pre-calculated using state-transition matrices, and are stored for all the possible 1022 chip-shift values [12]:

(7) $$M_{i}(n)={\left[\begin{array}{cc} {\vec{p}}_{i} & 1\\ \text{ID} & \vec{0} \end{array}\right]}^{n}\cdot M_{0}\;,$$

where *M_i_*(*n*) is the mask corresponding to the i_th_ sequence (*i* =1, 2) with a delay of *n* chips, *p⃗_i_* is a row vector containing the coefficients of the polynomial representing the *G_i_* LFSR (0010000001 for G_1_ and 0110010111 for G_2_), ID is the square 9-element identity matrix, 0⃗ is a 9-element zero's column vector and *M_0_* is the mask for a delay offset of 0 chips ( 0000000001). Thus, the *M_1_* and *M_2_* masks contain not only the C/A code delay offset value, but also the satellite ID to be generated. On the other hand, to allow for a code resolution smaller than one chip the block *resampler* was conceived (Fig. 5). Since the system's sampling frequency is 5.745 MHz and the period of the C/A code is 1 ms for 1023 chips, the relation ‘samples per chip’ is 5745/1023, which is equivalent to the irreducible fractional form of 1915/341. Therefore, a modulo-1915 counter increases in 341 units every clock cycle. When the counter “rolls-up” a pulse to drive the *G_1_* and *G_2_* LFSRs is issued (*pulse_CA* signal in Fig. 5). The initial value of this counter is related to the non-integer desired code delay. For instance, a non-integer chip value of 0.4 chips would correspond to an initial value of 0.4 x 1915 = 766 units.

Another capital core of the designed embedded system is the buffer for the reflected signal. Both the direct signal buffer and the reflected signal buffer share the same design (Fig. 1). It receives the multiplexed GPS data from PAU-RAD and demultiplexes it so that a DDM for a single beam can be processed at a time. This peripheral is composed of two storage units that alternatively switch their input and output ports. Whenever one is receiving and storing the I/Q data the other is being unloaded (one of the four beams at a time) to compute a new DDM. The clock frequency to receive the data bits is four times the sampling frequency of the data to allow the simultaneous storage of the data of four different beams. On the other hand, the unloading of the raw data and the generation of the DDM are performed at the clock frequency of the reflectometer (100 MHz), which is significantly higher than that of the incoming data (23 MHz). Thus again, hardware reuse is possible and up to four satellites can be processed in 1 ms. The switching between the storage units takes place whenever the one being written reaches its capacity limit (i.e, 5745 samples for each of the four beams), and an interrupt is issued by the buffer. This interrupt has top priority, since no data loss is allowed, and it triggers the generation of the DDMs associated to the data of the 1 ms under consideration. To achieve this, it is necessary to translate the delay and Doppler values of the signals received from different satellites to a set of parameters (the masks M1 and M2 of the LFSR's that determine the satellite ID and the C/A code offset, and the α values that determine the step of the frequency synthesizers that compensate the Doppler shifts), and send them to the DDM generator before the computation of a DDM.

**Figure 6.**
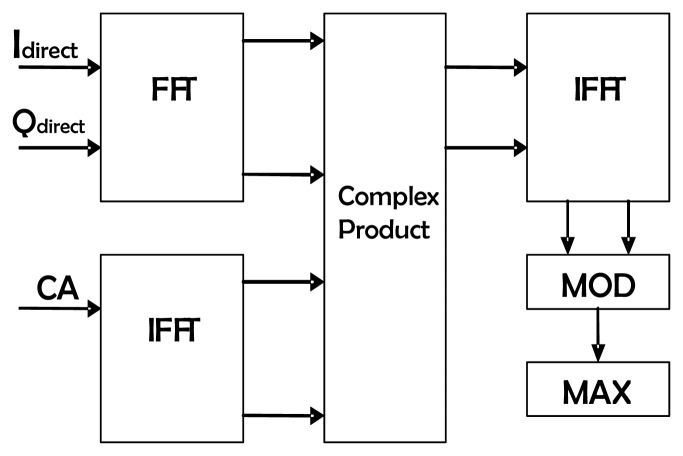
Architecture of the delay offset block.

**Figure 7.**
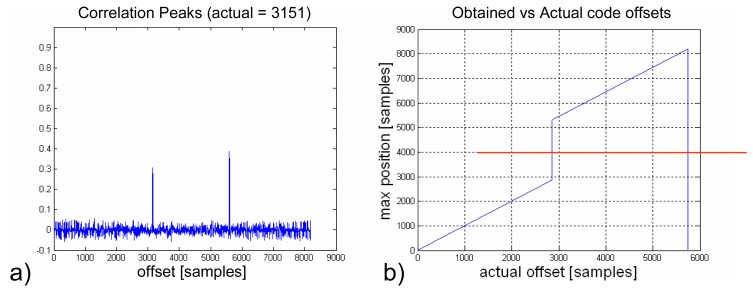
Correlation of the 5745-sample C/A code using standard FFT blocks with 2^13^ = 8192 input samples. (a) Two correlation maxima instead of a single one. (b) Relation of the actual code offset and the retrieved one.

In order to have the DDM maximum in the center of the window of delay and Doppler values it is necessary to have a good estimate of the delay and Doppler frequency of the received signal. At low and moderate altitudes the Doppler maximum value for both direct and reflected signals is roughly the same, and the difference lies only in the magnitude of the Doppler spread. Taking this into account, and also considering that the temporal derivative of these Doppler shifts (∼ 1 Hz/s [5]) is much smaller than the 1 s update rate of the parameters provided by the GPS receiver, its value can be used straightforwardly as the Doppler center value. Unfortunately the situation is quite the opposite when it comes to the delay value: it is necessary to estimate the delay difference between the direct and reflected signals from the transmitter-receiver geometry. For example, in the simplest case of a low altitude receiver at constant height *h* the excess delay is:

(8) $$\Delta \tau =\frac{2h}{c}\cos\theta \;,$$

being *θ* the zenith angle and *c* the light speed. This value must be added to the direct signal delay to obtain the reflected signal delay. However, the temporal derivative of the delay cannot be neglected over the 1 s update interval. Therefore, should the delay value obtained through the serial interface be used, the maximum of the DDM will move along the delay domain at a speed depending on the satellite's position until eventually getting out of view. This drift is very inconvenient, since integration is needed to improve the low SNR of the reflected signal. If the peak moves, the integration will only further degrade the waveform. Therefore it is of capital importance to have readily available an estimate for the delay updated the more frequently the better. To do so a whole new block is required to perform the circular correlation by means of Fast Fourier Transform operations to find the maximum of the correlation of the direct signal with clean CA code replicas for four different satellites simultaneously. The equation applied is [5]:

(9) $$R_{x,\text{ca}}=\text{IFFT}(\text{FFT}\left(x\right)\cdot \text{IFFT}\left(\text{ca}\right))\;,$$

where *x* is the baseband complex signal *I* + *j*·*Q*. Before computing such a correlation it is necessary to compensate the respective Doppler offsets by using the data provided by the uplooking GPS receiver and to generate for each of the four satellites a local replica with zero delay offset of their C/A codes. This block has been implemented with standard Fast Fourier Transform (FFT) and complex multiplier cores, ensuring its performance and preventing an excessive use of the FPGA resources (Fig. 6). Since the number of samples (5745 samples for 1 ms of sampled data) is not a power of 2, as needed to perform an FFT, it is necessary to fill the data values until reaching 8192 = 2ˆ13 samples. This results in two correlation maxima instead of only one. What is more, the amplitude of these maxima is position dependent. Fortunately these peaks are always spaced by the same amount of samples (8192-5745 = 2447 samples, as shown in Fig. 7a). Therefore, if the resulting value lays above a certain threshold, it would be necessary to subtracts from it those 2447 samples to obtain the actual offset value. If the value lays beneath the threshold, then it is already the value being searched (Fig. 7b).

### 2.2. Software Setup

The coordinated operation of all the peripherals that compose the reflectometer is ensured by the program running on the MicroBlaze soft processor. This code is written in ANSI C, and controls the GPS incoming data through the UART, the selection of the satellites, the generation of the DDM parameters and the signaling of the beginning of a new DDM computation. This program also transfers the DDM generation parameters to the DDM generator (the masks *M_1_* and *M_2_* of the LFSR's that determine the satellite ID and the C/A code offset, and the α values that determine the step of the frequency synthesizers that compensate the Doppler shifts). To do so these inputs are mapped to a RAM-like register at the DDM generator side. Then the read/write operations are performed by simply addressing to the DDM generator's address space. Another task of the code is the decoding of the data packets received from the commercial GPS receiver through the RS-232 link. These packets contain information on the available satellites and their respective delay, Doppler, power, elevation and azimuth values. From these values both the parameters to be fed to the DDM generator and the most suitable array beam for a given satellite are determined. Since it is another PAU system (PAU-RAD [11]) that is in charge for the beamforming, the beam selection is transmitted to it through a dedicated communication channel. To do so the elevation-azimuth space is divided according to a grid and a beam number is assigned to each region.

The program defines several constants such as the DDM resolution (step in both the delay and Doppler domains), the sampling frequency of the incoming data and a set of conversion factors (i.e., to convert from chips to samples). At the same time, the system variables and structures are also defined. They include the packet structures used to interact with the commercial GPS receiver, the masks of the LFSRs that generate the CA code with a certain delay, or a vector of the selected satellites to work with. The system functions can be grouped in three sets:

- The first one includes those that send and receive data from the GPS receiver,
- A second set selects which of the available satellites are to be used, taking into account their power and position, and finally
- Another set of critical functions computes the DDM parameters and transfers them to the DDM generator.

**Figure 8.**
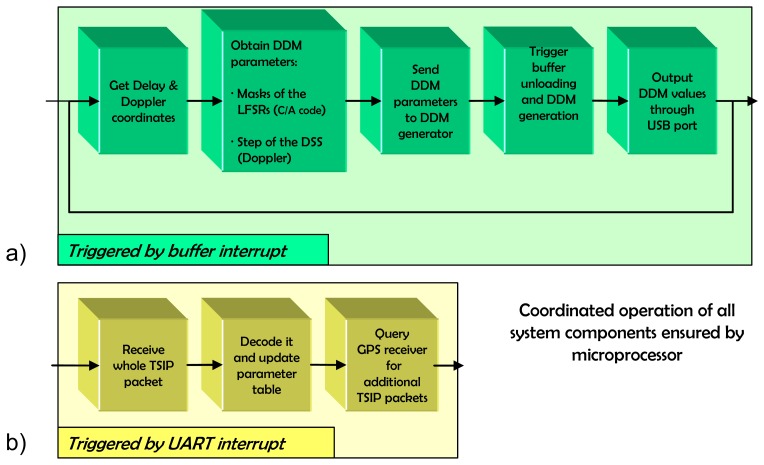
Interrupts and their associated Interrupt Service Routine (ISR) dataflow: (a) Buffer ISR and (b) UART ISR

The system has two sources of interrupts (Fig. 8). The buffer for the reflected signal (Fig. 1) issues an interrupt every 1 ms (i.e., whenever new data is available). This Interrupt Service Routine (ISR) plays an important role in the overall operation of the reflectometer, since the buffer interrupt is the synchronism reference of the system. It comprises the following tasks:

- First, the UART interrupts are disabled,
- Then, for each of the four simultaneous beams/satellites, the buffer is notified which beam has to dump next,
- The corresponding DDM parameters are set into the DDM generator,
- The generation of the DDM is triggered,
- The system waits until the *DDM_ready* signal goes high,
- Then the DDM data sending through the USB controller is enabled,
- The ISR waits for the USB controller to assert that the sending process is finished, and finally
- Before returning to the main function the UART interrupts are again enabled.

The RS-232 serial port controller issues an interrupt every time new data arrives from the GPS receiver:

- First of all, its associated ISR disables the UART interrupts,
- Then it reads from the UART buffer all the available data bytes and parses them to decode the TSIP report packets,
- Also, depending on the value of a counter, new Elevation & Azimuth packets are requested, and finally
- Before returning to the main program the interrupts for the UART are enabled.

The main program initializes the system and waits for interrupts:

- First, the UART interrupts are enabled in the RS-232 controller,
- Then a configuration command TSIP packet is sent to the GPS receiver, so that it outputs the raw data packet every 1 second,
- The satellite table is initialized and its associated DDM parameters are retrieved,
- The interrupts in the microprocessor and in the interrupt controller are enabled,
- The code waits for interrupts and, after an UART ISR has been executed, gets the new DDM parameters for the four simultaneous beams.

### 2.3. Data Output

**Figure 9.**
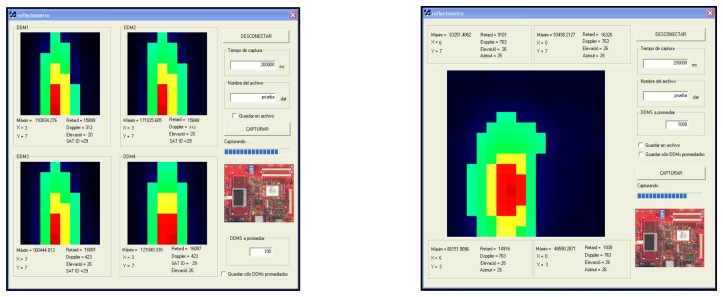
Implemented interface for receiving, displaying and storing the DDMs. On the left the 4-satellite operational mode is seen, whereas on the right the 1-satellite operational mode is shown. These videos correspond to actual measurements on the Garraf Cliffs (click to play them).

As it has already been pointed out, the computed DDM needs to be swiftly sent out of the FPGA system to be processed. This data link is divided in three areas. The first one is a peripheral synthesized in the FPGA that acts as an external master for the USB controller (CY7C68013 chip). Then there is the USB controller IC itself, composed of a microcontroller and an I/O buffer. The third section is the program running at the terminal computer. The FPGA master controller sends the computed DDM values to the I/O buffer, using additional control signals. It acts as a data bridge between the DDM generator and the USB controller, since it sweeps the output address range of the DDM generator, reading and sending data points away. The data interface is shown in Fig. 9. It has been written in Visual C++. After the program starts, it verifies that the USB controller is indeed connected to the computer through an USB cable and then uploads the firmware to it, so that it becomes configured. Additional control buttons allow configuring the integration times and the data files to store. So far two interfaces have been implemented, each of them associated to one operational mode. In the first one four satellites are tracked simultaneously, and up to 16 × 16 point DDMs can be displayed in real-time. The second mode operates with just one satellite, but combines the hardware resources so that its size is four times larger: 32 × 32.

## 3. Results

PAU-OR (one receiver) is a small-scale version of the complete PAU instrument [13]. It has been developed to test the concept before assembling the whole instrument, and also to allow more flexibility when trying to gather field data, because of its compactness. The reflectometer core, common to both instruments, has been assembled in PAU-OR to debug the reflectometer by acquiring real GPS data.

**Figure 10.**
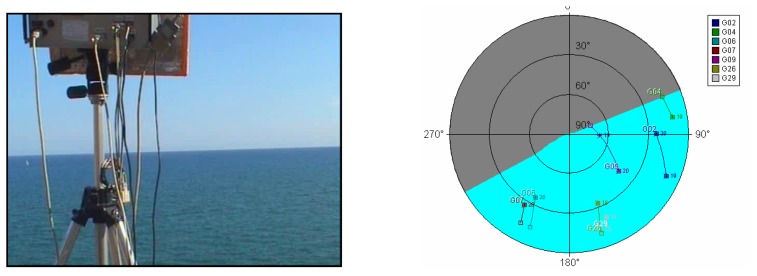
Experimental measurements at Garraf Cliffs. On the left PAU-OR points towards the sea surface from above the cliff. On the right, the position of the available satellites can be seen in a polar diagram. Results are shown in Fig. 9 for both modes of operation.

The experimental test shown took place at the Garraf cliffs, 30 km South of Barcelona. It was a calm sea day (SWH = 51 cm). To determine the GPS satellites with a suitable geometry for tracking their reflections (Fig. 10), a 45° incidence angle mask had to be considered due to the cliff steepness. The results are shown in Figs. 9a and 9b in both operational modes. The coherent integration time was set to 1 ms, and the averaged number of views (incoherent) was of 100. Depending on the surface under observation (land or sea, for example), the maximum coherent integration time must be selected according to the surface's correlation time, so that the coherent averaging of uncorrelated waveforms is not performed.

It is known that the shape of the DDMs associated to the signals reflected over a certain surface depends on the geometry of observation and on the nature of the surface: its roughness, spatial orientation, and dielectric permittivity.

## 4. Conclusions

An implementation of a real-time VHDL reflectometer has been presented. The design featured several in-house designed cores (DDM generator, delay offset estimator, buffer) to cope with the realtime nature of the system. There are three different data chains (reflected and direct signals and commercial GPS receiver data) that are combined together to obtain the system's observable: the delay-Doppler map (DDM). The shape of the DDMs can be linked to the Transmitter-Receiver geometry and to the scattering properties of the Earth's surface.

Future improvements of the system will include an improved interaction of the peripherals by means of direct links instead of the system bus. Regarding the measurements, field experiments will be performed during 2008 and 2009 to obtain collocated series of DDM's, brightness temperature data at L-band and sea state data (sea surface directional wave spectrum). It is expected that the full measurement of the DDMs will provide a more robust correlation with the brightness temperature change associated to the sea state, and thus will help improve the sea state correction required for accurate salinity retrieval.

## Footnotes

1 Existing instruments are able to provide in real-time just “waveforms” or cuts of the DDM at constant *f_d_*

2 For simplicity, the sign bit is retained after sampling at 8 bits, calibration and digital beamforming.

3 According to [5], the SNR decreases 1.96 dB and 0.55 dB alter 1-bit and 2-bit quantization, respectively.
